# Supplementary material for: Association of Air Pollutants with Incident Chronic Kidney Disease in a Nationally Representative Cohort of Korean Adults
Source: Int J Environ Res Public Health. 2021 Apr 4;18(7):3775. doi: 10.3390/ijerph18073775 (PMC8038583; doi:10.3390/ijerph18073775)
Supplement: Supplementary file 1 [file ijerph-18-03775-s001.pdf]

Table S1. Characteristics of the participants who received health examination according to the annual exposure to PM_{10}.

Participants who received health examination, n	Quartiles of Air Pollutants in annual average				Total
	First quartile	Second quartile	Third quartile	Forth quartile	
Age, years, n (%)					49,380
40-49	6,218 (44.74)	5,418 (42.23)	4,916 (43.79)	4,493 (39.33)	21,045 (42.6)
50-59	4,479 (32.23)	4,140 (32.27)	3,668 (32.67)	3,892 (34.07)	16,179 (32.8)
60-69	2,302 (16.56)	2,427 (18.92)	1,943 (17.31)	2,294 (20.08)	8,966 (18.2)
≥70	899 (6.47)	845 (6.59)	700 (6.23)	746 (6.53)	3,190 (6.5)
Sex, N (%)					
Men	7,320 (52.67)	6,424 (50.07)	5,709 (50.85)	5,714 (50.01)	25,167 (51.0)
Women	6,578 (47.33)	6,406 (49.93)	5,518 (49.15)	5,711 (49.99)	24,213 (49.0)
Body mass index, kg/m^2	23.8 (2.9)	23.8 (2.9)	23.9 (3.0)	23.9 (2.9)	23.9 (2.9)
Systolic blood pressure, mmHg	125.2 (17.4)	125.3 (17.5)	125.7 (17.6)	125.9 (17.5)	125.7 (17.5)
Fasting serum glucose, mg/dL	98.0 (27.1)	97.9 (29.1)	97.0 (26.1)	97.8 (28.2)	98.0 (28.2)
Total cholesterol, mg/dL	197.6 (37.2)	199.9 (37.2)	200.2 (37.7)	200.0 (37.5)	199.4 (37.4)
Alcohol consumption frequency, n (%)					
None	7,982 (57.4)	7,627 (59.5)	6,527 (58.1)	6,600 (57.8)	28,736 (58.2)
2-3 times per month	2,196 (15.8)	1,858 (14.5)	1,767 (15.7)	1,747 (15.3)	7,568 (15.3)
1-2 times per week	2,406 (17.3)	2,126 (16.6)	1,845 (16.4)	1,884 (16.5)	8,261 (16.7)
3-4 times per week	870 (6.3)	816 (6.4)	732 (6.5)	759 (6.6)	3,177 (6.4)
≥5 times per week	444 (3.2)	403 (3.1)	356 (3.2)	435 (3.8)	1,638 (3.3)
Physical activity, times per week, n (%)					
None	6,437 (46.3)	6,176 (48.1)	5,223 (46.5)	5,569 (48.7)	23,405 (47.4)
1-2	4,050 (29.1)	3,530 (27.5)	3,344 (29.8)	3,021 (26.4)	13,945 (28.2)
3-4	1,779 (12.8)	1,679 (13.1)	1,516 (13.5)	1,440 (12.6)	6,414 (13.0)
5-6	480 (3.5)	409 (3.2)	416 (3.7)	402 (3.5)	1,707 (3.5)
7	1,152 (8.3)	1,036 (8.07)	728 (6.5)	993 (8.7)	3,909 (7.9)
Smoking, n (%)					
Never	9,812 (70.6)	9,243 (72.0)	8,075 (71.9)	8,248 (72.2)	35,378 (71.6)
Former	769 (5.5)	606 (4.7)	674 (6.0)	574 (5.0)	2,623 (5.3)
Current	3,317 (23.9)	2,981 (23.2)	2,478 (22.1)	2,603 (22.8)	11,379 (23.0)
Charlson comorbidity index, n (%)					
0	4,630 (33.3)	4,411 (34.4)	4,105 (36.6)	3,946 (34.5)	17,092 (34.6)
1	3,882 (27.9)	3,654 (28.5)	3,152 (28.1)	3,180 (27.8)	13,868 (28.1)
≥2	5,386 (38.8)	4,765 (37.1)	3,970 (35.4)	4,299 (37.6)	18,420 (37.3)

Data are mean (SD) unless indicated otherwise.

Table S2. Sensitivity analysis on association of the air pollutants with incident chronic kidney disease using the first and last annual exposures.

Air pollutant	Quartiles of Air Pollutants in annual average				P_{trend}
	First quartile	Second quartile	Third quartile	Forth quartile	
**Annual exposure in 2002**					
PM_{10}, μg/m^3, range	42.8-61.2	64.9-72.9	73.6-77.1	77.4-101.7	
aHR (95% CI)	1.00 (reference)	1.01 (0.84-1.22)	0.95 (0.78-1.17)	0.92 (0.75-1.13)	0.240
SO_2, ppm, range	0.0024-0.0043	0.0044-0.0053	0.0053-0.0066	0.0066-0.0118	
aHR (95% CI)	1.00 (reference)	0.96 (0.83-1.12)	0.94 (0.80-1.10)	1.04 (0.87-1.25)	0.915
NO_2, ppm, range	0.0089-0.027	0.028-0.033	0.033-0.037	0.038-0.045	
aHR (95% CI)	1.00 (reference)	1.11 (0.90-1.38)	0.93 (0.73-1.20)	1.06 (0.84-1.35)	0.842
CO, ppm, range	0.23-0.61	0.62-0.70	0.71-0.74	0.74-1.01	
aHR (95% CI)	1.00 (reference)	0.98 (0.85-1.14)	1.01 (0.86-1.17)	1.03 (0.89-1.20)	0.622
O_3, ppm, range	0.011-0.014	0.014-0.016	0.016-0.020	0.020-0.030	
aHR (95% CI)	1.00 (reference)	1.08 (0.93-1.25)	1.03 (0.87-1.23)	1.21 (0.99-1.49)	0.138
**Annual exposure in 2005**					
PM_{10}, μg/m^3, range	38.0-51.4	51.7-55.7	56.5-62.3	62.5-74.7	
aHR (95% CI)	1.00 (reference)	0.97 (0.84-1.13)	0.97 (0.84-1.13)	1.07 (0.92-1.24)	0.378
SO_2, ppm, range	0.0036-0.0044	0.0045-0.0056	0.0056-0.0065	0.0067-0.0130	
aHR (95% CI)	1.00 (reference)	0.99 (0.85-1.15)	1.09 (0.94-1.26)	1.06 (0.90-1.25)	0.234
NO_2, ppm, range	0.014-0.024	0.024-0.029	0.029-0.033	0.034-0.048	
aHR (95% CI)	1.00 (reference)	1.12 (0.96-1.31)	1.08 (0.89-1.31)	1.04 (0.83-1.29)	0.907
CO, ppm, range	0.28-0.50	0.51-0.57	0.58-0.71	0.72-1.28	
aHR (95% CI)	1.00 (reference)	1.00 (0.86-1.16)	0.95 (0.82-1.09)	0.97 (0.81-1.16)	0.538
O_3, ppm, range	0.013-0.017	0.017-0.020	0.020-0.022	0.022-0.031	
aHR (95% CI)	1.00 (reference)	1.21 (1.02-1.43)	1.16 (1.00-1.34)	1.13 (0.94-1.34)	0.301

aHR calculated by Cox proportional hazards regression after adjustments for age, sex, insurance premium, area of residence, and Charlson comorbidity index.

Acronyms: PM, particulate matter; aHR, adjusted hazard ratio; CI, confidence interval; SO_2, sulfur dioxide; NO_2, nitrogen dioxide; CO, carbon monoxide; O_3, ozone.

Table S3. Association of the air pollutants with incident chronic kidney disease among Korean adults aged at least 40 years.

Air pollutant	Quartiles of Air Pollutants in annual average				P_{trend}
	First quartile	Second quartile	Third quartile	Forth quartile	
PM_{10}, μg/m^3, range	37.3-58.1	58.1-62.9	63.4-65.3	65.4-81.5	
aHR (95% CI)[a]	1.00 (reference)	1.05 (0.86-1.28)	1.06 (0.87-1.30)	1.03 (0.84-1.26)	0.920
aHR (95% CI)[b]	1.00 (reference)	0.95 (0.76-1.19)	0.97 (0.77-1.22)	0.96 (0.76-1.21)	0.974
aHR (95% CI)[c]	1.00 (reference)	0.97 (0.74-1.28)	1.04 (0.79-1.38)	1.07 (0.81-1.42)	0.831
SO_2, ppm, range	0.0035-0.0045	0.0045-0.0054	0.0054-0.0062	0.0064-0.0120	
aHR (95% CI)[a]	1.00 (reference)	1.07 (0.92-1.25)	0.99 (0.84-1.18)	1.08 (0.88-1.33)	0.691
aHR (95% CI)[b]	1.00 (reference)	1.08 (0.90-1.29)	0.99 (0.82-1.21)	1.00 (0.79-1.27)	0.757
aHR (95% CI)[c]	1.00 (reference)	1.04 (0.84-1.28)	0.99 (0.79-1.25)	1.04 (0.78-1.38)	0.970
NO_2, ppm, range	0.015-0.027	0.027-0.032	0.032-0.037	0.037-0.043	
aHR (95% CI)[a]	1.00 (reference)	0.98 (0.79-1.21)	0.94 (0.74-1.20)	0.99 (0.77-1.27)	0.927
aHR (95% CI)[b]	1.00 (reference)	0.92 (0.72-1.18)	0.93 (0.71-1.23)	0.89 (0.67-1.19)	0.865
aHR (95% CI)[c]	1.00 (reference)	0.98 (0.73-1.31)	0.93 (0.67-1.30)	0.86 (0.61-1.23)	0.779
CO, ppm, range	0.28-0.58	0.58-0.64	0.66-0.68	0.69-0.84	
aHR (95% CI)[a]	1.00 (reference)	1.02 (0.87-1.19)	1.04 (0.89-1.22)	0.99 (0.83-1.16)	0.923
aHR (95% CI)[b]	1.00 (reference)	1.06 (0.88-1.27)	1.03 (0.86-1.25)	0.98 (0.81-1.19)	0.877
aHR (95% CI)[c]	1.00 (reference)	1.01 (0.81-1.26)	1.04 (0.84-1.30)	1.00 (0.79-1.26)	0.979
O_3, ppm, range	0.012-0.014	0.014-0.018	0.018-0.021	0.021-0.030	
aHR (95% CI)[a]	1.00 (reference)	1.07 (0.92-1.24)	1.08 (0.87-1.32)	1.05 (0.80-1.37)	0.835
aHR (95% CI)[b]	1.00 (reference)	1.06 (0.89-1.26)	1.04 (0.82-1.33)	0.99 (0.72-1.36)	0.876
aHR (95% CI)[c]	1.00 (reference)	1.02 (0.83-1.26)	1.05 (0.79-1.40)	0.89 (0.61-1.29)	0.622

aHR calculated by Cox proportional hazards regression after adjustments for age, sex, insurance premium, area of residence, and Charlson comorbidity index.

[a]calculated after washing out 1 year of latent period.

[b]calculated after washing out 3 years of latent period.

[c]calculated after washing out 5 years of latent period.

Acronyms: PM, particulate matter; aHR, adjusted hazard ratio; CI, confidence interval; SO_2, sulfur dioxide; NO_2, nitrogen dioxide; CO, carbon monoxide; O_3, ozone.

Table S4. Sensitivity analysis on association of the air pollutants with incident chronic kidney disease after additional 3 years of air pollutant exposures.

Air pollutant	Quartiles of Air Pollutants in annual average				P_{trend}
	**First quartile**	**Second quartile**	**Third quartile**	**Forth quartile**	
PM_{10}, μg/m^3, range	44.6-56.9	57.6-60.7	61.1-63.7	63.9-71.3	
aHR (95% CI)	1.00 (reference)	1.00 (0.81-1.24)	1.12 (0.90-1.40)	1.03 (0.84-1.27)	0.403
SO_2, ppm, range	0.0038-0.0049	0.0051-0.0055	0.0055-0.0066	0.0066-0.0146	
aHR (95% CI)	1.00 (reference)	0.99 (0.86-1.13)	1.01 (0.87-1.17)	0.95 (0.80-1.13)	0.922
NO_2, ppm, range	0.013-0.026	0.026-0.033	0.033-0.037	0.038-0.042	
aHR (95% CI)	1.00 (reference)	1.00 (0.83-1.19)	1.00 (0.79-1.27)	0.99 (0.78-1.25)	0.998
CO, ppm, range	0.30-0.57	0.57-0.60	0.61-0.65	0.66-0.79	
aHR (95% CI)	1.00 (reference)	1.14 (0.97-1.35)	1.09 (0.93-1.28)	1.01 (0.85-1.21)	0.276
O_3, ppm, range	0.014-0.016	0.016-0.018	0.018-0.021	0.021-0.030	
aHR (95% CI)	1.00 (reference)	0.96 (0.84-1.10)	1.02 (0.86-1.20)	1.19 (0.85-1.66)	0.636

aHR calculated by Cox proportional hazards regression after adjustments for age, sex, insurance premium, area of residence, and Charlson comorbidity index.

Acronyms: PM, particulate matter; aHR, adjusted hazard ratio; CI, confidence interval; SO_2, sulfur dioxide; NO_2, nitrogen dioxide; CO, carbon monoxide; O_3, ozone.
